# Hypermethylation of the glutathione peroxidase 4 promoter predicts poor prognosis in patients with hepatitis B virus-associated acute-on-chronic liver failure

**DOI:** 10.3389/fmolb.2024.1421597

**Published:** 2024-07-25

**Authors:** Xing Su, Li-Yan Han, Jing Wang, Ying Zhang, Peng-Yu Luo, Shuai Gao, Yu-Chen Fan, Jing-Wei Wang, Kai Wang

**Affiliations:** ^1^ Department of Hepatology, Qilu Hospital of Shandong University, Jinan, Shandong, China; ^2^ Hepatology Institute of Shandong University, Jinan, Shandong, China; ^3^ Department of Hepatology, Qilu Hospital (Qingdao) of Shandong University, Qingdao, Shandong, China

**Keywords:** glutathione peroxidase 4, HBV-associated acute-on-chronic liver failure, prognosis, biomarker, DNA methylation

## Abstract

**Background:**

Hepatitis B virus-associated acute-on-chronic liver failure (HBV-ACLF) is a syn-drome with a high short-term mortality rate, and its prognosis is critical in clinical management. This study aimed to investigate the clinical significance of glutathione peroxidase 4 (GPX4) in the occurrence and development of HBV-ACLF and its prognostic value for 90-day mortality.

**Methods:**

The expression levels of GPX4, oxidative stress-related molecules and inflammatory cytokines in serum or peripheral blood mononuclear cells (PBMCs) of 289 participants were determined by RT-qPCR or ELISA, and the methylation level of GPX4 promoter in PBMCs was determined by MethyLight.

**Results:**

The expression levels of GPX4 in the PBMCs and serum of HBV-ACLF patients were lower than those in non-HBV-associated acute-on-chronic liver failure (non-HBV ACLF) patients, patients with chronic hepatitis B (CHB) and healthy control (HC) individuals, while the methylation level of the *GPX4* promoter was greater. In HBV-ACLF patients, the methylation level of the *GPX4* promoter is correlated with oxidative stress, inflammation-related molecules, and some clinicopathological indicators. The methylation level of the *GPX4* promoter was identified as an independent risk factor for 90-day mortality in HBV-ACLF patients and yielded a larger area under the receiver operating characteristic curve (AUROC) than the model for end-stage liver disease (MELD) score in predicting 90-day mortality.

**Conclusion:**

The GPX4 promoter methylation level has promising potential as a predictor of 90-day mortality in patients with HBV-ACLF.

## 1 Introduction

Acute-on-chronic liver failure (ACLF) is a clinical syndrome of acute decompensation of liver function based on chronic liver disease; characterized by intense systemic inflammation, organ failure, and a poor prognosis (Arroyo et al., 2020). Unlike in Western countries, hepatitis B virus (HBV) infection remains a critical cause of ACLF in Asian countries. It accounts for ∼70% of ACLF cases in Asian countries, and alcohol-related ACLF accounts for as little as 15%. HBV-ACLF can rapidly progress to multiorgan failure within 4 weeks, with high mortality and poor prognosis (Sarin et al., 2019). Thus, early and accurate prediction of prognosis is essential for life-saving emergent treatment.

GPX4 is a unique antioxidant enzyme that protects cells from membrane lipid peroxidation and maintains redox homeostasis, by reducing highly reactive lipid hydroperoxides (LOOHs) to nonreactive lipid alcohols (Yang et al., 2014; Xie et al., 2023). Redox homeostasis is the balance of oxidation and reduction reactions present in all living systems and is a crucial component of physiological cell homeostasis. Impaired redox homeostasis is associated with multiple pathological conditions, including viral diseases and cancer (Ursini et al., 2016; Agmon and Stockwell, 2017; Lennicke and Cochemé, 2021). Oxidative stress (OS) plays a significant role in the pathogenesis of liver failure (Zaccherini et al., 2021; Piano et al., 2023). If the antioxidant capacity is insufficient and/or the damage incurred is not efficiently repaired, reactive oxygen species (ROS) may ultimately lead to OS-related damage, such as cell apoptosis, tissue damage, and organ dysfunction which is the pathophysiological basis for the development and progression of ACLF (Engelmann et al., 2021). Extensive studies have shown that GPX4 is involved in the occurrence and development of a variety of inflammatory diseases and liver diseases (Xie et al., 2023), but the potential significance and prognostic value of GPX4 in HBV-ACLF patients have not been determined.

In this study, we primarily used MethyLight to detect the methylation levels of *GPX4* promoter in patients with HBV-ACLF, patients with non-HBV ACLF, patients with CHB and HCs. We subsequently analyzed the relationships between the methylation level of the *GPX4* promoter and oxidative damage indicators, inflammatory cytokines, and clinicopathological indicators in HBV-ACLF patients to clarify the potential clinical significance of GPX4 in the occurrence and development of HBV-ACLF and to determine its potential as a predictor of 90-day mortality in HBV-ACLF patients.

## 2 Materials and methods

### 2.1 Study population

**FIGURE 1:**
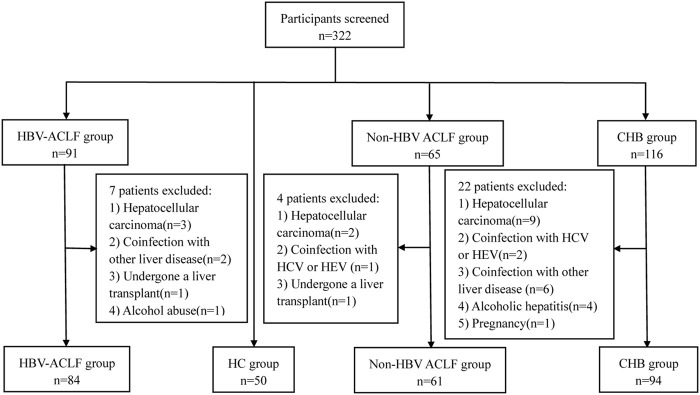
The selection process of subjects.

A total of 289 participants were recruited from September 2021 through September 2023 in the Department of Hepatology, Qilu Hospital of Shandong University including 84 patients with HBV-ACLF, 61 patients with non-HBV ACLF, 94 patients with CHB and 50 HCs (Figure 1). HBV-ACLF patients and non-HBV ACLF patients were defined according to the criteria proposed by the Asian Pacific Association for the Study of the Liver (APASL) (Sarin et al., 2019). The inclusion criteria of HBV-ACLF patients were HBV infection for at least 6 months, jaundice (serum total bilirubin [TBIL] > 85 μmol/L), abnormal coagulation (international normalized ratio [INR]≥1.5 or prothrombin activity [PTA] < 40%), and ascites and/or hepatic encephalopathy (HE) within 4 weeks. The subjects with the following situation will be excluded: coinfection with hepatitis A, C, D, and E virus, HIV and other viruses; combined with other liver diseases, such as autoimmune hepatitis, alcoholic hepatitis, drug hepatitis; combined with pregnancy, metabolic disorders, primary liver cancer and other malignant tumors; combined with serious diseases of other systems; patients with severe hepatitis caused by HBV reinfection after liver transplantation; rejected participants. The inclusion criteria of Non-HBV ACLF patients were jaundice (TBIL > 85 μmol/L), abnormal coagulation (INR≥1.5 or PTA < 40%), and ascites and/or HE within 4 weeks. The subjects with the following situation will be excluded: coinfection with hepatitis A, C, D, and E virus, HIV and other viruses; combined with pregnancy; combined with primary liver cancer; combined with other malignant tumors; combined with serious diseases of other systems; patients after liver transplantation; rejected participants. Patients with CHB were diagnosed based on hepatitis B surface antigen (HBsAg) positivity for > 6 months according to the American Association for the Study of Liver Disease (AASLD) (Terrault et al., 2018). Healthy volunteers served as normal controls with negative viral hepatitis tests, normal alanine aminotransferase (ALT)/aspartate aminotransferase (AST) levels, and no evidence of other liver or malignant diseases. This research was reviewed and approved by the Medical Ethical Committee of Qilu Hospital of Shandong University and conducted according to the Declaration of Helsinki. All participants signed informed consent after understanding the experimental process and required specimens.

After enrollment, the basic information, clinical data, and laboratory parameters were recorded. According to the APASL consensus recommendations, patients with HBV-ACLF were treated with standard therapies, such as antiviral treatment, liver protection and other necessary management methods, such as plasma exchange, according to the individual assessment. None of the patients with ACLF in this study underwent liver transplantation. All patients with HBV-ACLF were followed for 3 months after the start of the study.

### 2.2 Serum collection and PBMCs isolation

Peripheral venous blood (3 mL) was collected from each participant into an EDTA-containing tube following an 8 h fast and whole blood (5 mL) was also collected in additional serum separator tubes (SST) for serum isolation. Following centrifugation, the serum was isolated and PBMCs were obtained through Ficoll-Paque Plus (GE Healthcare, Uppsala, Sweden) density gradient centrifugation.

### 2.3 DNA extraction and TaqMan probe-based quantitative methylation-specific PCR

Genomic DNA was extracted from PBMCs using TRIzol Reagent (Invitrogen, Carlsbad, CA, United States). EZ DNA Methylation-Gold kit (Zymoresearch, Orange, CA, United States) was used for DNA bisulfite modification. MethyLight was performed using the EpiTect MethyLight PCR + ROX Vial Kit (QIAGEN, Hilden, Germany) and according to the manufacturer’s standard guidelines. We used the website (https://www.ncbi.nlm.nih.gov/) to delineate the promoter of *GPX4*. The genome coordinates of *GPX4* are hg38, chr19: 1103994-1106779. We selected the upstream 2,000 bp region of its transcription start site (TSS) as the promoter region. Then, we used another website (https://www.urogene.org/methprimer/) for sequence transformation and found only one CpG island (from 1,678 bp to 1,945 bp) through it (Li and Dahiya, 2002). So, the primers and probes were designed at this unique CpG island region (Supplementary Figure S1) through the Oligo7 (OLIGO 1267 Vondelpark ColoradoSprings, CO 80907, United States). Two sets of primers and probes, specifically designed for bisulfite-converted DNA, were utilized for the assay and listed in Table 1: an experimental set for the *GPX4* gene and a reference set for the *ACTB* gene, which served as a normalization control. The percentage of the methylation reference value (PMR) indicates MethyLight data (Gao et al., 2015).

$$\text{PMR}=100\%\times 2^{-\left[∆\text{Ct}\;\left(\text{target}\;\text{gene}\;-\;\text{control}\;\text{gene}\right)\;\text{Sample}\;-∆\text{Ct}\;\left(\text{target}\;\text{gene}\;-\;\text{control}\;\text{gene}\right)\;M.\text{SssI}-\text{Reference}\right]}$$

**TABLE 1:** Sequences of used primers and probes*.*

	Gene	Forward primer sequence (5’ to 3’)	Reverse primer sequence (5’ to 3’)	Probe sequence (5’ to 3’)
RT-qPCR	GPX4	AAGTAAACTACACTCAGCTCGTC	AAACCACACTCAGCGTATCGG	
	ACTB	CATGTACGTTGCTATCCAGGC	CTCCTTAATGTCACGCACGAT	
MethyLight	GPX4	TCGACGGGTATATGGTTAATTTGGAT	GTTAATAACGATACACACGAAACCCCTA	CCAAACGAACGCCCACCGAT
	ACTB	TGGTGATGGAGGAGGTTTAGTAAGT	AACCAATAAAACCTACTCCTCCCTTAAA	ACCACCACCCAACACACAATAACAAACACA

Probe modification: 5’6-FAM and 3’BHQ1.

### 2.4 RNA extraction and RT-qPCR

Total RNA was extracted from PBMCs using TRIzol Reagent (Invitrogen, Carlsbad, CA, United States) and complementary DNA (cDNA) was then immediately synthesized using the First-Strand cDNA Synthesis Kit (Fermentas, Vilnius, Lithuania) according to the manufacturer’s instructions. The cDNA was used as a template immediately for RT-qPCR. The 10 μL reaction system underwent denaturation at 95°C for 30 s, followed by 45 cycles of 95°C for 5 s, 60°C for 30 s and 72°C for 60 s. The sequences of specific primers for *GPX4* and *ACTB* were both described in Table 1. The relative expression of *GPX4* was calculated using the 2^−ΔΔCT^ method. All amplification reactions were conducted in triplicate.

### 2.5 Enzyme-linked immunosorbent assay (ELISA)

Serum cytokine levels were quantified using the Human Immunoassay Valukine ELISA Kits for GPX4, tumor necrosis factor α (TNF-α), interleukin-6 (IL-6), interleukin-1β (IL-1β), ROS, malonaldehyde (MDA) and superoxide dismutase (SOD) (Lengton Bioscience Co., Shanghai, China). These kits employ a competitive method to detect samples’ content, and absorbance was measured at 450 nm according to the manufacturer’s protocol. All samples were measured in triplicate.

### 2.6 Clinical parameters

The serum biochemical markers included ALT, AST, TBIL, albumin (ALB), and creatinine (Cr). Blood routine indicators included white blood cell (WBC) counts and platelet (PLT) counts. Hemostasis markers included prothrombin time (PT), INR values, and PTA. Markers of viral infection included HBsAg, hepatitis B e antigen (HBeAg), and HBV DNA. These markers were measured using operating procedures in the Department of Medicine Laboratory, Qilu Hospital of Shandong University. Model for end-stage liver disease (MELD) score was calculated according to the original formula (Malinchoc et al., 2000):

$$\begin{array}{l} \text{MEL}D\text{ score}=\left[9.57\times \ln\left(\text{creatinine}\left[\text{mg}/\text{dL}\right]\right)+3.78\times \ln\left(\text{bilirubin}\left[\text{mg}/\text{dL}\right]\right)\right.\\ \;\left.+|11.20|\times ||\ln|\left(\text{INR}\right)|+|6.43|\left(\text{constant for liver disease etiology}:\right.\right.\\ \;\left.\left.0\text{ if cholestatic or alcoholic};\;1\text{ otherwise}\right)\right]. \end{array}$$

### 2.7 Statistical analysis

Statistical analyses were performed using SPSS version 26.0 statistical software (SPSS Inc., Chicago, IL, United States). Quantitative variables are expressed as median (centile 25; centile 75). Categorical variables were expressed as numbers (%). Mann-Whitney U test, Kruskal–Wallis Test and Dunn’s test were used to compare the quantitative variables. The chi-square test was used to compare the categorical variables. The Spearman’s rank correlation test was used to analyze the relationship between *GPX4* methylation level and quantitative clinical data as well as *GPX4* mRNA expression level, serum GPX4, TNF-α, IL-6, IL-1β, ROS, MDA, and SOD levels. Receiver operating characteristic (ROC) curves were generated to estimate the discriminations. Survival analysis was performed using the Kaplan–Meier method with the log-rank test. All statistical analyses were 2-sided, and *p*-value < 0.05 was considered statistically significant.

## 3 Results

### 3.1 Baseline characteristics of the study populations

In this study, a total of 289 participants were enrolled, including 84 patients with HBV-ACLF, 61 patients with non-HBV ACLF, 94 patients with CHB and 50 HCs. The general characteristics, clinical manifestations, and laboratory measurements of the subjects are shown in Table 2.

**TABLE 2:** General clinical characteristics of the patients.

Variables	HBV-ACLF (n = 84)	Non-HBV ACLF (n = 61)	CHB(n = 94)	HCs(n = 50)	*P*-value
Male, n (%)	64 (76.19)	39 (63.93)	53 (56.38)	29 (58.00)	0.036^a^
Age (years)	49 (40–58)	52 (46–62)	47 (37–55)	52 (39–58)	0.052 ^b^
log10 [HBV-DNA]	4.59 (3.17–5.81)	NA	6.90 (4.73–8.19)	NA	<0.001^b^
ALT (U/L)	131.00 (64.50–350.75)	50.00 (26.50–116.00)	42.00 (20.00–115.00)	16.00 (13.00–22.50)	<0.001 ^b^
AST (U/L)	119.00 (79.75–234.25)	67.00 (43.00–156.00)	30.00 (20.00–61.00)	19.00 (16.00–23.00)	<0.001 ^b^
TBIL (µmol/L)	244.40 (165.70–359.88)	226.30 (160.30–360.25)	12.20 (8.58–16.40)	11.20 (8.25–14.45)	<0.001 ^b^
ALB (g/L)	33.35 (30.00–36.73)	31.90 (30.35–34.35)	46.25 (44.18–49.25)	46.95 (44.98–48.40)	<0.001 ^b^
Cr (µmol/L)	57.00 (48.0–64.75)	48.00 (38.00–71.50)	69.00 (54.75–79.00)	70.00 (64.75–79.25)	<0.001 ^b^
WBC(10^9/L)	7.16 (5.35–9.88)	7.34 (5.35–10.97)	5.61 (4.84–6.79)	5.77 (4.72–6.85)	< .001 ^b^
PLT (10^9/L)	96.00 (66.00–135.00)	103.00 (63.00–169.50)	210.50 (178.00–243.25)	233.00 (198.00–274.50)	<0.001 ^b^
PTA (%)	38.00 (29.00–47.00)	43.00 (31.00–49.00)	107.00 (94.00–114.00)	112.00 (104.00–121.00)	<0.001 ^b^
INR	1.96 (1.59–2.41)	1.79 (1.60–2.33)	0.96 (0.92–1.04)	0.93 (0.89–0.97)	<0.001 ^b^
AFP (ng/mL)	62.20 (15.88–218.50)	4.63 (3.37–11.90)	3.05 (2.05–5.95)	3.03 (2.44–4.55)	<0.001 ^b^
MELDs	20.50 (16.50–23.78)	15.48 (10.27–18.98)	NA	NA	<0.001^c^
Cirrhosis, n (%)	48 (57.14)	44 (72.13)	0	0	0.064^a^
Ascites, n (%)	46 (54.76)	39 (63.93)	0	0	0.268^a^
HE, n (%)	17 (20.24)	9 (14.75)	0	0	0.395^a^

Quantitative variables were expressed as medians (25th, 75th percentage).

Qualitative variables were expressed as numbers (percentages).

^a^ Chi-square test.

^b^ Kruskal–Wallis H test.

^c^ Mann–Whitney U test.

The baseline characteristics of the HBV-ACLF patients were categorized as survivors or nonsurvivors based on the 90-day prognosis, as shown in Table 3. Of the 84 HBV-ACLF patients consecutively enrolled, 29 were nonsurvivors, with a median age of 52 years (47–60 years). Compared to the survivors, the nonsurvivors exhibited significantly lower PLT counts, PTA and *GPX4* relative mRNA expression levels, but significantly higher TBIL levels, WBC counts, INR values, MELD scores, rates of HE and *GPX4* PMR values (all *p* < 0.05).

**TABLE 3:** Baseline characteristics of patients with HBV-ACLF stratified by 90-day prognosis.

Variables	Survival (n = 55)	Non-survival (n = 29)	*P*-value
Male, n (%)	44 (80.00)	20 (68.97)	0.259^a^
Age (years)	46 (38–54)	52 (47–60)	0.057 ^b^
log10 [HBV-DNA]	4.02 (3.05–5.90)	4.86 (3.46–5.66)	0.392 ^b^
HBsAg(IU/mL)	1,251.96 (192.82–4387.25)	4,297.51 (249.97–6,383.50)	0.228 ^b^
HBeAg(+), n (%)	39 (70.91)	17 (58.62)	0.256^a^
ALT (U/L)	114.00 (64.00–363.00)	140.00 (69.00–271.00)	0.818 ^b^
AST (U/L)	114.00 (79.00–239.00)	125.00 (82.00–194.50)	0.563 ^b^
TBIL (µmol/L)	191.70 (142.30–312.00)	339.60 (240.65–497.95)	<0.001^b^
ALB (g/L)	33.60 (28.80–37.10)	33.30 (30.60–35.80)	0.682 ^b^
Cr (µmol/L)	57.00 (48.00–62.00)	56.00 (48.00–75.50)	0.379 ^b^
WBC (10^9/L)	6.14 (5.14–9.80)	8.00 (6.53–11.53)	0.032 ^b^
PLT (10^9/L)	102.00 (74.00–149.00)	77.50 (58.50–119.50)	0.048 ^b^
PTA (%)	44.00 (37.00–49.00)	29.00 (25.00–35.00)	<0.001^b^
INR	1.68 (1.55–2.01)	2.40 (2.17–2.77)	<0.001^b^
AFP (ng/mL)	80.54 (15.65–320.40)	51.46 (15.73–136.64)	0.414 ^b^
MELDs	17.59 (14.91–20.92)	22.93 (20.81–26.60)	<0.001^b^
Cirrhosis, n (%)	28 (50.91)	20 (68.97)	0.112^a^
Ascites, n (%)	26 (47.27)	20 (68.97)	0.058^a^
HE, n (%)	5 (9.09)	12 (41.38)	<0.001^a^
*GPX4* relative mRNA expressive	0.25 (0.22–0.26)	0.21 (0.19–0.23)	0.001^b^
*GPX4* PMR (%)	79.00 (74.09–90.75)	102.81 (90.56–130.15)	<0.001^b^

Quantitative variables were expressed as medians (25th, 75th percentage).

Qualitative variables were expressed as numbers (percentages).

^a^chi-square test.^b^ Mann–Whitney U test.

### 3.2 Hypermethylation of the *GPX4* promoter in HBV-ACLF patients

**FIGURE 2:**
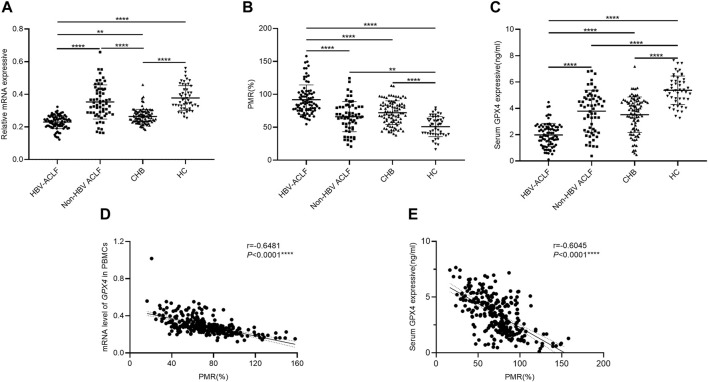
The expression patterns of GPX4 in different groups. **(A)** Relative mRNA levels of *GPX4* in PBMCs from HBV-ACLF patients, non-HBV ACLF patients, CHB patients and HCs. **(B)** *GPX4* methylation levels in PBMCs from HBV-ACLF patients, non-HBV ACLF patients, CHB patients and HCs. **(C)** Serum GPX4 levels in HBV-ACLF patients, non-HBV ACLF patients, CHB patients and HCs. **(D)** A significant correlation was observed between the PMR value of the *GPX4* promoter and the mRNA level in PBMCs (Spearman’s r = −0.6481, *P*< 0.0001). **(E)** A significant correlation was observed between the PMR value of *GPX4* promoter and serum GPX4 expression (Spearman’s r = −0.6045, *p* < 0.0001), ns, *p* > 0.05; *, *p* ≤ 0.05; **, *p* ≤ 0.01; ***, *p* ≤ 0.001; ****, *p* ≤ 0.0001.

To investigate the potential involvement of GPX4, we initially examined the expression level of *GPX4* and the methylation status of the *GPX4* promoter, expressed as the PMR value, in the enrolled study population. First, we analyzed the relative mRNA level of *GPX4* in PBMCs from HBV-ACLF patients, non-HBV ACLF patients, CHB patients and HCs (Figure 2A). Compared to that in HCs, the relative mRNA level of *GPX4* was significantly lower in HBV-ACLF (*p* < 0.0001) and CHB (*p* < 0.0001) patients respectively. Moreover, the relative mRNA level of *GPX4* was significantly lower in the HBV-ACLF patients than in the non-HBV ACLF patients (*p* < 0.0001) and CHB patients (*p* = 0.0030). Next, we analyzed the methylation status of the *GPX4* promoter expressed as the PMR values in PBMCs from patients with HBV-ACLF, non-HBV ACLF, CHB and HCs (Figure 2B). Compared to that in HCs, the *GPX4* promoter methylation level was significantly greater in HBV-ACLF patients (*p* < 0.0001), non-HBV ACLF patients (*p* = 0.0014) and CHB patients (*p* < 0.0001) respectively. Moreover, the *GPX4* promoter methylation level was significantly greater in HBV-ACLF patients than in non-HBV ACLF patients (*p* < 0.0001) and CHB patients (*p* < 0.0001). In addition, we analyzed the serum GPX4 levels in HBV-ACLF patients, non-HBV ACLF patients, CHB patients and HCs (Figure 2C). Compared to that in HCs, the serum GPX4 level was significantly lower in HBV-ACLF patients (*p* < 0.0001), non-HBV ACLF patients (*p* < 0.0001) and CHB patients (*p* < 0.0001) respectively. And the serum GPX4 level was significantly lower in HBV-ACLF patients than in non-HBV ACLF (*p* < 0.0001) and CHB patients (*p* < 0.0001).

The relationships between *GPX4* methylation levels and mRNA levels in PBMCs and between *GPX4* methylation levels and serum expression levels in all study populations were further analyzed by using Spearman rank correlation analysis. We found that the PMR value of *GPX4* was significantly negatively correlated with the mRNA level of *GPX4* in PBMCs (Spearman’s r = −0.6481, *P*< 0.0001) and the serum GPX4 expression level (Spearman’s r = −0.6045, *P*< 0.0001), as shown in Figures 2D, E.

### 3.3 Associations between the *GPX4* promoter methylation levels and the serum expression levels of related cytokines in HBV-ACLF patients

**FIGURE 3:**
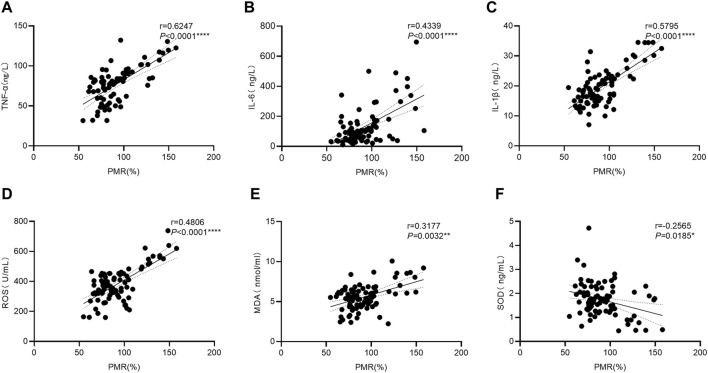
Associations between the *GPX4* promoter methylation level and the serum expression levels of related cytokines in HBV-ACLF patients. Significant correlations were observed between the PMR value of *GPX4* promoter and serum TNF-α expression (**(A)** Spearman’s r = 0.6247, *P*< 0.0001), and serum IL-6 expression (**(B)** Spearman’s r = 0.4339, *P*< 0.0001), and serum IL-1β expression (**(C)** Spearman’s r = 0.5795, *P*< 0.0001). Significant correlations were observed between the PMR value of *GPX4* promoter and serum ROS expression (**(D)** Spearman’s r = 0.4806, *P*< 0.0001), and serum MDA expression (**(E)** Spearman’s r = 0.3177, *p* = 0.0032), and serum SOD expression (**(F)** Spearman’s r = −0.2565, *p* = 0.0185), ns, *p* > 0.05; *, *p* ≤ 0.05; **, *p* ≤ 0.01; ***, *p* ≤ 0.001; ****, *p* ≤ 0.0001.

To further explore the mechanism(s) underlying the increase in *GPX4* promoter methylation levels in HBV-ACLF patients, we examined the serum expression levels of cytokines associated with OS, antioxidant stress, and inflammation in patients with HBV-ACLF by using ELISA. The relationships between *GPX4* promoter methylation levels and these cytokines were further analyzed by using the Spearman rank correlation test, and the results are shown in Figure 3. As shown in the figure, the PMR value of *GPX4* was significantly positively correlated with the serum TNF-α concentration (Figure 3A Spearman’s r = 0.6247, *p* < 0.0001), the serum IL-6 concentration (Figure 3B Spearman’s r = 0.4339, *p* < 0.0001), the serum IL-1β concentration (Figure 3C Spearman’s r = 0.5795, *p* < 0.0001), the serum ROS concentration (Figure 3D Spearman’s r = 0.4806, *p* < 0.0001) and the serum MDA concentration (Figure 3E Spearman’s r = 0.3177, *p* = 0.0032). Additionally, the PMR value of *GPX4* was significantly negatively correlated with the serum SOD concentration (Figure 3F Spearman’s r = −0.2565, *p* = 0.0185).

### 3.4 Associations between the GPX4 promoter methylation levels and clinicopathological features in HBV-ACLF patients

**FIGURE 4:**
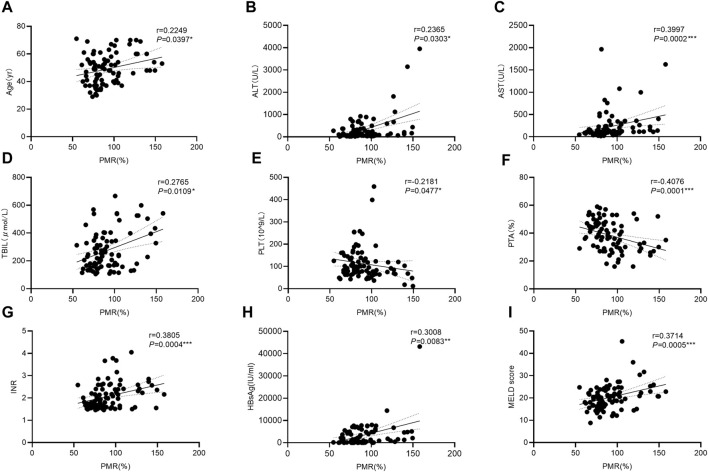
Associations between the *GPX4* methylation level and clinical variables in HBV-ACLF patients. Significant correlations were observed between the PMR value of *GPX4* promoter and Age (**(A)** Spearman’s r = 0.2249, *P* = 0.0397), and ALT (**(B)** Spearman’s r = 0.2365, *p* = 0.0303), and AST (**(C)** Spearman’s r = 0.3997, *p* = 0.0002), and TBIL (**(D)** Spearman’s r = 0.2765, *p* = 0.0109), and PLT (**(E)** Spearman’s r = 0.2181, *p* = 0.0477), and PTA (**(F)** Spearman’s r = 0.4076, *p* < 0.0001), and INR (**(G)** Spearman’s r = 0.3805, *p* = 0.0004), and HBsAg (**(H)** Spearman’s r = 0.3008, *p* = 0.0083), and MELD score (**(I)** Spearman’s r = 0.3714, *p* = 0.0005), ns, *p* > 0.05; *, *p* ≤ 0.05; **, *p* ≤ 0.01; ***, *p* ≤ 0.001; ****, *p* ≤ 0.0001.

To further explore the clinical significance of the hypermethylation of the *GPX4* promoter, we identified that the *GPX4* methylation level was positively correlated with age (Spearman’s r = 0.2249, *P* = 0.0397), ALT (Spearman’s r = 0.2365, *p* = 0.0303), AST (Spearman’s r = 0.3997, *p* = 0.0002), TBIL (Spearman’s r = 0.2765, *p* = 0.0109), INR (Spearman’s r = 0.3805, *p* = 0.0004), HBsAg (Spearman’s r = 0.3008, *p* = 0.0083) and MELD scores (Spearman’s r = 0.3714, *p* = 0.0005), and negatively correlated with PLT (Spearman’s r = −0.2181, *P* = 0.0477) and PTA (Spearman’s r = −0.4076, *p* < 0.0001) (Figures 4A–I). However, it was not associated with HBV-DNA, HBeAg, ALB, WBC and AFP (all *p* > 0.05).

In addition, the HBV-ACLF patients were divided into several subgroups according to their basic characteristics and clinicopathological features as shown in Table 4. We found that the *GPX4* methylation level was significantly greater in patients with cirrhosis, patients with ascites, patients with HE and patients with MELD score > 20. However, the *GPX4* promoter methylation level was not significantly correlated with gender and AFP level.

**TABLE 4:** Associations between clinicopathological features and GPX4 promoter methylation level.

Variables	Number	PMR (%)	*P*-value
Gender			
Male	64	84.67 (76.58–102.81)	0.610
Female	20	93.95 (75.88–101.22)	
Age (years)			
≤50	47	84.09 (77.38–99.31)	0.254
>50	37	92.66 (75.26–111.91)	
log10 [HBV-DNA]			
≤3	18	83.54 (77.52–100.18)	0.905
>3	66	86.46 (75.66–102.81)	
AFP (ng/mL)			
≤20	22	91.71 (76.05–103.35)	0.741
>20	62	84.64 (76.18–100.87)	
Cirrhosis			
Positive	48	93.31 (77.38–107.77)	0.023
Negative	36	80.94 (72.27–93.52)	
Ascites			
Positive	46	93.95 (78.60–111.78)	0.003
Negative	38	79.83 (71.08–90.02)	
HE			
Positive	17	96.59 (85.00–123.60)	0.021
Negative	67	83.51 (75.26–99.31)	
MELDs			
≤20	40	79.00 (74.38–93.95)	0.002
>20	44	93.96 (81.23–115.56)	

Quantitative variables were expressed as medians (25th, 75th percentage).

### 3.5 Hypermethylation of the GPX4 promoter served as an independent risk factor for 90-day mortality of HBV-ACLF patients

**FIGURE 5:**
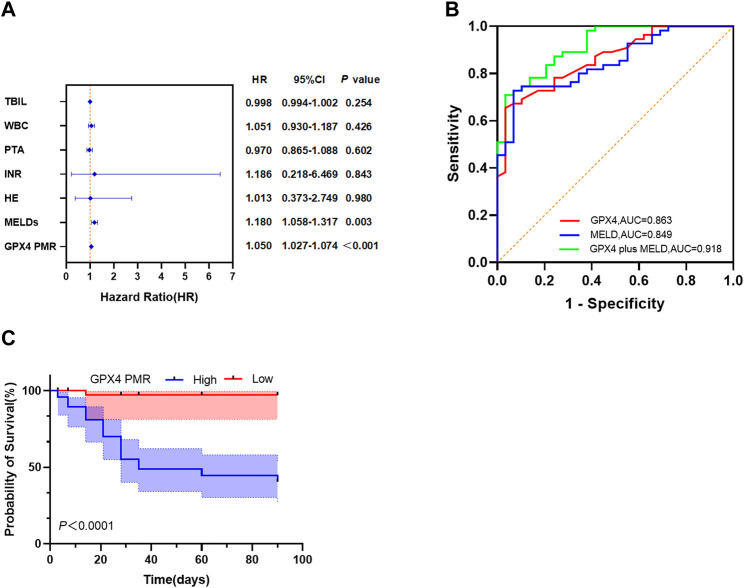
GPX4 PMR outperforms the MELD score as a biomarker in HBV-ACLF patients. **(A)** Multivariate Cox proportional hazard regression analyses of prognostic factors associated with 90-day mortality in patients with HBV-ACLF. **(B)** ROC curves of the GPX4 PMR, MELDs and GPX4 PMR plus MELDs for predicting 90-day mortality in patients with HBV-ACLF. The PMR values of the GPX4 promoter had an AUC of 0.863, which was greater than that of the MELDs (AUC = 0.849). The optimal cut-off point of 62.00% was selected (cut-off PMR = 83.80%). The GPX4 PMR plus MELDs had an AUC of 0.918, which was greater than that of the GPX4 PMR and MELDs. **(C)** Kaplan-Meier curves for patients with HBV-ACLF stratified according to the GPX4 methylation level.

We performed univariate and multivariate Cox proportional hazard regression analyses to identify factors associated with the clinical outcomes in HBV-ACLF patients. As shown in Table 5, the TBIL level (HR = 1.004, 95%CI: 1.002–1.007), WBC count (HR = 1.121, 95%CI: 1.009–1.246), PTA (HR = 0.910, 95%CI: 0.875–0.946), INR (HR = 3.465, 95%CI: 2.124–5.652), HE (HR = 3.419, 95%CI: 1.627–7.182), MELD score (HR = 1.217, 95%CI: 1.141–1.299) and *GPX4* PMR (HR = 1.050, 95%CI: 1.034–1.067) were significantly correlated with 90-day mortality in HBV-ACLF patients. Multivariate Cox proportional hazard analysis revealed that the *GPX4* PMR (HR = 1.050, 95%CI:1.027–1.074) and MELD score (HR = 1.180, 95%CI:1.058–1.317) were independently associated with the prognosis of HBV-ACLF patients (Figure 5A).

**TABLE 5:** Univariate and multivariate Cox proportional hazard regression analyses of prognosticators associated with 90-day mortality in patients with HBV-ACLF.

Variables	Univariate analysis		Univariate analysis	
	HR (95%CI)	P-value	HR (95%CI)	P-value
Gender (female vs. male)	0.649 (0.295–1.426)	0.282		
Age (years) (>50 vs. ≤ 50)	1.612 (0.775–3.352)	0.201		
log10 [HBV-DNA] (high vs. low)	1.082 (0.887–1.321)	0.435		
HBsAg (IU/mL) (high vs. low)	1.000 (1.000–1.000)	0.876		
HBeAg (positive vs. negative)	1.857 (0.886–3.890)	0.101		
ALT (U/L) (high vs. low)	1.000 (1.000–1.001)	0.570		
AST (U/L) (high vs. low)	1.000 (0.999–1.001)	0.629		
TBIL (µmol/L) (high vs. low)	1.004 (1.002–1.007)	<0.001	0.998 (0.994–1.002)	0.254
ALB (g/L) (high vs. low)	1.018 (0.945–1.097)	0.643		
WBC (10^9/L) (high vs. low)	1.121 (1.009–1.246)	0.034	1.051 (0.930–1.187)	0.426
PLT (10^9/L) (high vs. low)	0.994 (0.987–1.001)	0.115		
PTA (%) (high vs. low)	0.910 (0.875–0.946)	<0.001	0.970 (0.865–1.088)	0.602
INR (high vs. low)	3.465 (2.124–5.652)	<0.001	1.186 (0.218–6.469)	0.843
Cirrhosis (positive vs. negative)	1.279 (0.617–2.649)	0.508		
Ascites (positive vs. negative)	1.137 (0.549–2.357)	0.729		
HE (positive vs. negative)	3.419 (1.627–7.182)	0.001	1.013 (0.373–2.749)	0.980
MELDs (high vs. low)	1.217 (1.141–1.299)	<0.001	1.180 (1.058–1.317)	0.003
GPX4 PMR (high vs. low)	1.050 (1.034–1.067)	<0.001	1.050 (1.027–1.074)	<0.001

Quantitative variables were expressed as medians (25th, 75th percentage).

### 3.6 Diagnostic value of the GPX4 promoter methylation level for predicting 90-day mortality of HBV-ACLF patients

We employed ROC curves to evaluate the diagnostic value of *GPX4* promoter methylation levels for predicting 90-day mortality in patients with HBV-ACLF (Figure 5B). The value of the top left corner of the ROC curve, with the maximum sum of sensitivity and specificity, was determined as the optimal diagnostic cut-off value. As shown in Figure 5B; Table 6, when predicting the 90-day mortality of HBV-ACLF patients, the optimal *GPX4* PMR cut-off value (83.80%) was used to predict survival and nonsurvival, and an AUC of 0.863 (95%CI: 0.787–0.940) was achieved, with a sensitivity of 65.5% and a specificity of 96.6%. The MELD score allowed us to discriminate between survivors and nonsurvivors at an optimal cut-off value of 20.50, with an AUC of 0.849 (95%CI: 0.769–0.929), sensitivity of 72.7%, and specificity of 93.1%. The combination of the *GPX4* PMR value and MELD score yielded an AUC of 0.918 (95%CI: 0.861–0.975), with a sensitivity of 70.9% and a specificity of 96.6%. The results suggested that the *GPX4* PMR achieved a significantly greater AUC than did the MELD score. Notably, this combination had greater prognostic accuracy for HBV-ACLF than did the MELD score alone or the *GPX4* PMR alone. Hence, the *GPX4* PMR is a better biomarker for predicting 90-day mortality of HBV-ACLF patients than the MELD score.

**TABLE 6:** Diagnostic values of GPX4 PMR, MELD score and GPX4 PMR plus MELD score for predicting 90-day mortality of patients with HBV-ACLF.

Parameter	Sensitivity (%)	Specificity (%)	Youden index	AUC	95%CI
GPX4 PMR	65.5	96.6	0.620	0.863	0.787–0.940
MELD	72.7	93.1	0.658	0.849	0.769–0.929
GPX4 PMR plus MELD	70.9	96.6	0.675	0.918	0.861–0.975

The median survival time for patients with high *GPX4* PMR values was 35 days. Due to the limited sample size, we could not determine the median survival time for patients with low *GPX4* PMR values. We then evaluated the prognostic performance of the *GPX4* PMR in patients with HBV-ACLF. Kaplan–Meier analysis implied that hypermethylation of the *GPX4* promoter was associated with worse overall survival (*p* < 0.001 by log-rank test, Figure 5C).

## 4 Discussion

HBV-ACLF can manifest at any stage of CHB. Liver function rapidly deteriorates due to various factors; jaundice and coagulation dysfunction are the primary clinical manifestations, with ascites encephalopathy appearing within 4 weeks. HBV-ACLF is one of the most common end-stage liver diseases and is a life-threatening clinical syndrome (Piano et al., 2023). Approximately 12,000 patients with CHB per year are estimated to die from HBV-ACLF in Asia. Given its acute onset, rapid progression, poor prognosis, limited medical treatment options, scarcity of liver donors for transplantation, and high cost, early identification and prognostic prediction of this disease are crucial (Bernal et al., 2015; Hsu et al., 2023). To this end, a large number of researchers have established prognostic models based on the pathological process of ACLF, using various clinical indicators that can reflect liver function, organ failure, infection and inflammation (Liu et al., 2020; Nie et al., 2020; Xue et al., 2021). However, these models have not been widely used in clinical practice due to various limitations such as complex operation and high cost. Therefore, it is still of great significance to explore the pathological mechanism of HBV-ACLF and to find more accurate biomarkers that are easy to be applied in clinic.

Recent studies have shown that the pathophysiological mechanisms of ACLF mainly include a severe systemic inflammatory response, immune dysfunction, mitochondrial dysfunction, metabolic changes and OS (Engelmann et al., 2021). Due to the differences in the basis and causes of chronic liver disease, the pathogenesis of ACLF is significantly different between Eastern and Western populations. In the Asia-Pacific region, ACLF is mostly caused by HBV reactivation or overlapping infection with other hepatotropic viruses (Sarin and Choudhury, 2016). The pathogenesis of HBV-ACLF is extremely complex and is considered the result of interactions among multiple factors, such as the environment, virus and host. However, the exact mechanism has not been elucidated. It is generally believed that immune cell-mediated inflammation is the core link in the pathogenesis of HBV-ACLF, and OS is also involved. Due to immune disorders mediated by HBV infection and cytotoxic effects, cell death and tissue damage caused by OS cause an imbalance in proinflammatory cytokines, which further promotes the progression of HBV-ACLF.

GPX4, a glutathione peroxidase, is a key regulator of the ferroptosis regulatory pathway. GPX4 protects cells from the accumulation of lipid peroxides and reduces OS damage by scavenging free radicals and participating in the hydrolysis of lipid peroxides. It is an antioxidant molecule (Li et al., 2018). Research has shown that GPX4 is involved in numerous biological functions, including neuronal loss, autophagy, cell repair, inflammation, ferroptosis, apoptosis, and OS(Weaver and Skouta, 2022). Inhibition of GPX4, either directly or indirectly, leads to insufficient clearance of lipid peroxides, resulting in the accumulation of these harmful substances within cells. This accumulation then leads to OS damage and triggers ferroptosis. Ferroptosis is a recently discovered form of iron-dependent programmed cell death that is distinct from other known types of cell death such as apoptosis, necrosis, and autophagy. The main mechanisms underlying ferroptosis involve an imbalance in the amino acid antioxidant system, disruption of iron metabolism, and the accumulation of lipid peroxides (Jiang et al., 2021). This process is consistent with the imbalance of iron homeostasis and oxidative damage observed during the occurrence and development of HBV-ACLF; therefore, we speculate that ferroptosis may be involved in the pathological process of HBV-ACLF. Furthermore, damage-associated molecular patterns (DAMPs) released by cells after ferroptosis can trigger inflammatory responses through various pathways, including the Toll-like receptor 4 (TLR4) pathway, the advanced glycosylation end product-specific receptor (AGER) pathway, and the stimulator of interferon response cGAMP interactor 1 (STING1) pathway (Chen et al., 2021), which further supports our hypothesis. However, whether ferroptosis is indeed involved in this process remains to be investigated. In addition, GPX4 loss mediates the cross-linking of ferroptosis with oxidative stress, inflammation/pyroptosis, and autophagy (Xie et al., 2023). These pathological processes may be involved in hepatocyte death and specific immune cell depletion in the development of HBV-ACLF (Lan et al., 2017; Wang F., et al., 2021).

Recent studies have shown that GPX4 plays a significant role in the development and progression of various diseases. In particular, it has been implicated in inflammatory diseases, autoimmune disorders, neurodegenerative diseases, ischemia-reperfusion (I/R) injury, and cancers. Regarding inflammatory diseases, experiments in mouse models have shown that the conditional depletion of *GPX4* in myeloid cells expedites the systemic inflammatory response and leads to multiorgan failure (Kang et al., 2018). Additionally, GPX4 inhibits inflammatory nuclear factor-κB (NF-κB) pathway activation and the production of proinflammatory mediators (e.g., IL1B and PTGS2/[prostaglandin-endoperoxide synthase 2] [COX2]) across diverse experimental models, such as angiogenesis and hair follicle development (Banning et al., 2004; Sengupta et al., 2013). GPX4 deficiency in the pancreas or small intestine can lead to pancreatitis (Liu et al., 2022) and inflammatory bowel disease (Mayr et al., 2020). Collectively, these models involving conditional depletion of *GPX4* suggest a broad regulatory role for GPX4 in the prevention of inflammation. Notably, GPX4 also plays a crucial role in the occurrence and progression of liver diseases. It has been established that GPX4 is indispensable for the survival of hepatocytes and for the normal functioning of the liver (Carlson et al., 2016). Therefore, targeting GPX4 may be a potential therapeutic approach for a variety of liver diseases. For example, inhibiting GPX4 activity has been shown to induce ferroptosis in hepatocellular carcinoma (HCC) cells (Wang Q. et al., 2021), and targeting GPX4 can alleviate ferroptosis and aid in the treatment of metabolism-related fatty liver disease (Tong et al., 2022). The activation of GPX4 by Maresin1 (MaR1) has been found to alleviate liver injury and reduce the expression of ROS, MDA, and inflammatory factors (Yang W. Wang, et al., 2022). Although GPX4 has been extensively studied in a wide range of diseases, there is currently no research on its role in HBV-ACLF. Considering that *GPX4* is widely expressed in immune cells and is the first line of defense of the immune system against inflammation, we conducted experiments using PBMCs to evaluate *GPX4* expression. Additionally, previous studies have shown that impaired expression of the *GPX4* gene in the PBMCs of breast cancer patients serves as a biomarker for an increased risk of breast cancer (Bermano et al., 2010), further suggesting the association between *GPX4* expression in PBMCs and disease.

One category of epigenetic modifications is DNA methylation, a chemical modification of DNA. DNA methylation is the acquisition of methyl groups by DNA methyltransferase (DNMT), which in turn affects gene expression without changing the DNA sequence. DNA methylation mainly occurs on the cytosines adjacent to guanine (CpG dinucleotide). DNA methylation in the gene promoter region can inhibit gene expression and lead to decreased gene expression levels (Jones, 2012). DNA methylation is frequently observed in the context of chronic inflammatory diseases such as cholangitis, inflammatory bowel disease, hepatitis, and liver fibrosis (Wei et al., 2023; Xu et al., 2023). Moreover, some studies have shown that abnormal expression of DNMTs is related to persistent HBV infection (Fan et al., 2016). Additionally, hepatitis B virus protein X (HBx) is known to induce various forms of epigenetic modifications, such as H3K4 methylation and DNA hypermethylation (Gao et al., 2020; Yang L. et al., 2022), indicating that abnormal methylation of the gene promoter occurs in individuals with HBV infection. Therefore, we hypothesized that DNA methylation may be involved in the development of HBV-ACLF. More importantly, the methylation test has higher stability than the common index test, which is more suitable for clinical application in the diagnosis and prediction of the disease, and the abnormal methylation status of genes in PBMCs has been used to diagnose and predict the prognosis of the diseases (Sun et al., 2018; Sun et al., 2022; Li et al., 2023). However, there is currently no available research investigating the methylation status of the *GPX4* promoter in PBMCs from HBV-ACLF patients and its potential association with prognosis.

Therefore, based on the pathophysiologic mechanism of HBV-ACLF, the biological function of GPX4, and the impact of GPX4 in other diseases, we speculate that GPX4 may be involved in multiple pathophysiological processes of HBV-ACLF and ultimately affect the overall prognosis of HBV-ACLF patients, and may be able to serve as an important biomarker for it. To verify this hypothesis, we initially found that GPX4 expression was significantly downregulated and that promoter methylation was significantly upregulated in HBV-ACLF patients compared with non-HBV ACLF patients, CHB patients and HC individuals. These results suggest that a decrease in GPX4 expression is caused by hypermethylation in the PBMCs of HBV-ACLF patients. The difference between HBV-ACLF and non-HBV ACLF patients suggested that HBV infection may have an effect on *GPX4* expression, and this phenomenon of viral influence on *GPX4* expression also occurs during Epstein-Barr virus infection (Yuan et al., 2022). In addition, Jia et al., (2020) found that GPX4 maintained reoxidation-reduction homeostasis and promoted STING-mediated innate immune response. In their study, the innate immune response induced by HSV-1 infection in GPX4-deficient/inactivated mice was significantly lower than that in the control group, and the virus replication and tissue damage were significantly higher than that in the control group. STING signaling pathway plays an important role in the course of chronic HBV infection, so it can be speculated that GPX4 influences HBV virus replication and tissue damage caused by STING signaling pathway, thus affecting the disease progression of HBV-ACLF. In our study, the etiology of Non-HBV ACLF group was complex and diverse, including drug-induced liver disease, alcoholic liver disease, etc., and its pathological process was also different. Therefore, the GPX4 expression levels and the methylation levels of *GPX4* gene promoter in PBMCs were significantly different in the Non-HBV ACLF group, but the differences were not significant compared with CHB patients or HCs, which is worthy of further study. Besides, we detected the levels of OS-related indicators and proinflammatory cytokines in the serum of HBV-ACLF patients. We found that the *GPX4* methylation level was significantly negatively correlated with the serum SOD level and significantly positively correlated with the serum ROS, MDA, TNF-α, IL-1β and IL-6 levels. This is consistent with the induction of OS and inflammation when GPX4 is inhibited. We then analyzed the relationship between *GPX4* promoter methylation levels and viral load in the PBMCs of HBV-ACLF patients and found some positive correlations between them. We speculate that HBV infection may inhibit the expression of *GPX4*, and in related studies, a decrease in *GPX4* expression was observed in primary hepatocytes transduced with lenti-HBx (Liu et al., 2021). However, this speculation needs to be verified by further studies. The *GPX4* methylation level was also positively correlated with liver injury indicators, such as AST and ALT, indicating that GPX4 is related to hepatocyte injury and the expression level of GPX4 can reflect the degree of liver injury in HBV-ACLF patients to a certain extent. Taken together, these results support the hypothesis that *GPX4* plays an important role in the pathogenesis of HBV-ACLF.

Given the correlation between GPX4 and oxidative damage indexes, inflammation indexes and liver clinicopathological indexes in HBV-ACLF patients, which can reflect the degree of oxidative damage, inflammation and hepatocyte damage in HBV-ACLF patients to a certain extent, and is suitable to be used as a biomarker for the prediction of prognosis in them, we evaluated the prognostic predictive potential of GPX4 in HBV-ACLF. Through univariate and multivariate Cox regression analyses, we found that the *GPX4* promoter methylation level was an independent risk factor for predicting 90-day mortality in HBV-ACLF patients. In terms of the predictive value of prognosis, the predictive ability of the *GPX4* PMR was greater than that of the MELD score, and the combination of the two further enhanced the predictive ability. According to the clinical follow-up data of HBV-ACLF patients, the overall survival of patients with *GPX4* promoter hypomethylation (PMR < 83.80%) was better than that of patients with *GPX4* promoter hypermethylation (PMR ≥ 83.80%). Overall, GPX4 has important clinical value in HBV-ACLF disease. Notably, the methylation status of the *GPX4* promoter was detected only in PBMCs and not in liver tissue, mainly because of the difficulty in obtaining liver tissue specimens from HBV-ACLF patients. In addition, PBMCs, which include monocytes, T cells, B cells, natural killer cells and dendritic cells, are important cells of the immune system and play key roles in the body’s inflammation and immune deterioration. Previous studies have reported that various diseases can affect the gene expression of PBMCs through host immunity or the inflammatory response. And Li et al.,(2022) have revealed that the expression of some genes in PBMCs can reflect the immune metabolism disorder of HBV-ACLF through PBMCs transcriptomic identification. Moreover, PBMCs are easy to obtain, and studies of PBMCs combined with serum cytokines may reveal the role of some of the body’s immune system in the occurrence and development of diseases.

This study has several limitations. First, the sample size was relatively small, and all patients were from a single center, which may have led to selection bias. Thus, the predictive power of the *GPX4* PMR needs to be validated prospectively in a larger sample. Second, we evaluated only the correlation between GPX4 and cytokine expression levels and between GPX4 and clinicopathological indicators and did not explore the underlying molecular mechanism. Future studies should aim to investigate the underlying molecular pathways through which GPX4 is involved in the development of HBV-ACLF. In conclusion, we found that the *GPX4* promoter is hypermethylated in patients with HBV-ACLF. This hypermethylation pattern was shown to be correlated with OS-related indicators and proinflammatory cytokine levels. These findings suggest that GPX4 may play an important role in the pathogenesis of HBV-ACLF and may provide new ideas for the development of comprehensive treatment strategies for this disease. Furthermore, we demonstrated that the *GPX4* PMR can accurately predict 90-day mortality in HBV-ACLF patients. Our results highlight the important role and potential prognostic value of *GPX4* in managing this disease, but the exact role of *GPX4* in the pathogenesis of HBV-ACLF and its potential as a therapeutic target need to be further investigated.

## 5 Conclusion

GPX4 may play an important role in the pathogenesis of HBV-ACLF, and hypermethylation of the *GPX4* promoter predicts poor prognosis in patients with HBV-ACLF, which will lead to new ideas for the clinical management of HBV-ACLF.

## Data Availability

The original contributions presented in the study are included in the article/Supplementary Material, further inquiries can be directed to the corresponding author.

## Supplementary material

SUPPLEMENTARY FIGURE S1
The CpG island in the GPX4 gene promoter region.

## References

[B1] Agmon E. Stockwell B. R. (2017). Lipid homeostasis and regulated cell death. Curr. Opin. Chem. Biol. 39, 83–89. 10.1016/j.cbpa.2017.06.002 28645028 PMC5581689

[B2] Arroyo V. Longo D. L. Moreau R. Rajiv J. (2020). Acute-on-Chronic liver failure. N. Engl. J. Med. 382 (22), 2137–2145. 10.1056/NEJMra1914900 32459924

[B3] Banning A. Schnurr K. Böl G. F. Kupper D. Müller-Schmehl K. Viita H. (2004). Inhibition of basal and interleukin-1-induced VCAM-1 expression by phospholipid hydroperoxide glutathione peroxidase and 15-lipoxygenase in rabbit aortic smooth muscle cells. Free Radic. Biol. Med. 36 (2), 135–144. 10.1016/j.freeradbiomed.2003.10.027 14744625

[B4] Bermano G. Smyth E. Goua M. Heys S. D. Wahle K. W. (2010). Impaired expression of glutathione peroxidase-4 gene in peripheral blood mononuclear cells: a biomarker of increased breast cancer risk. Cancer Biomark. 7 (1), 39–46. 10.3233/cbm-2010-0146 21045263 PMC12922868

[B5] Bernal W. Jalan R. Quaglia A. Simpson K. Wendon J. Burroughs A. (2015). Acute-on-chronic liver failure. Lancet 386 (10003), 1576–1587. 10.1016/s0140-6736(15)00309-8 26423181

[B6] Carlson B. A. Tobe R. Yefremova E. Tsuji P. A. Hoffmann V. J. Schweizer U. (2016). Glutathione peroxidase 4 and vitamin E cooperatively prevent hepatocellular degeneration. Redox Biol. 9, 22–31. 10.1016/j.redox.2016.05.003 27262435 PMC4900515

[B7] Chen X. Kang R. Kroemer G. Tang D. (2021). Ferroptosis in infection, inflammation, and immunity. J. Exp. Med. 218 (6), e20210518. 10.1084/jem.20210518 33978684 PMC8126980

[B8] Engelmann C. Clària J. Szabo G. Bosch J. Bernardi M. (2021). Pathophysiology of decompensated cirrhosis: portal hypertension, circulatory dysfunction, inflammation, metabolism and mitochondrial dysfunction. J. Hepatol. 75 (Suppl 1), S49–s66. 10.1016/j.jhep.2021.01.002 34039492 PMC9272511

[B9] Fan X. P. Ji X. F. Li X. Y. Gao S. Fan Y. C. Wang K. (2016). Methylation of the glutathione-S-transferase P1 gene promoter is associated with oxidative stress in patients with chronic hepatitis B. Tohoku J. Exp. Med. 238 (1), 57–64. 10.1620/tjem.238.57 26725685

[B10] Gao S. Sun F. K. Fan Y. C. Shi C. H. Zhang Z. H. Wang L. Y. (2015). Aberrant GSTP1 promoter methylation predicts short-term prognosis in acute-on-chronic hepatitis B liver failure. Aliment. Pharmacol. Ther. 42 (3), 319–329. 10.1111/apt.13271 26040771

[B11] Gao W. Jia Z. Tian Y. Yang P. Sun H. Wang C. (2020). HBx protein contributes to liver carcinogenesis by H3K4me3 modification through stabilizing WD repeat domain 5 protein. Hepatology 71 (5), 1678–1695. 10.1002/hep.30947 31544250

[B12] Hsu Y. C. Huang D. Q. Nguyen M. H. (2023). Global burden of hepatitis B virus: current status, missed opportunities and a call for action. Nat. Rev. Gastroenterol. Hepatol. 20 (8), 524–537. 10.1038/s41575-023-00760-9 37024566

[B13] Jia M. Qin D. Zhao C. Chai L. Yu Z. Wang W. (2020). Redox homeostasis maintained by GPX4 facilitates STING activation. Nat. Immunol. 21 (7), 727–735. 10.1038/s41590-020-0699-0 32541831

[B14] Jiang X. Stockwell B. R. Conrad M. (2021). Ferroptosis: mechanisms, biology and role in disease. Nat. Rev. Mol. Cell Biol. 22 (4), 266–282. 10.1038/s41580-020-00324-8 33495651 PMC8142022

[B15] Jones P. A. (2012). Functions of DNA methylation: islands, start sites, gene bodies and beyond. Nat. Rev. Genet. 13 (7), 484–492. 10.1038/nrg3230 22641018

[B16] Kang R. Zeng L. Zhu S. Xie Y. Liu J. Wen Q. (2018). Lipid peroxidation drives gasdermin D-mediated pyroptosis in lethal polymicrobial sepsis. Cell Host Microbe 24 (1), 97–108. 10.1016/j.chom.2018.05.009 29937272 PMC6043361

[B17] Lan P. Fan Y. Zhao Y. Lou X. Monsour H. P. Zhang X. (2017). TNF superfamily receptor OX40 triggers invariant NKT cell pyroptosis and liver injury. J. Clin. Invest 127 (6), 2222–2234. 10.1172/jci91075 28436935 PMC5451219

[B18] Lennicke C. Cochemé H. M. (2021). Redox metabolism: ROS as specific molecular regulators of cell signaling and function. Mol. Cell 81 (18), 3691–3707. 10.1016/j.molcel.2021.08.018 34547234

[B19] Li C. Deng X. Xie X. Liu Y. Friedmann Angeli J. P. Lai L. (2018). Activation of glutathione peroxidase 4 as a novel anti-inflammatory strategy. Front. Pharmacol. 9, 1120. 10.3389/fphar.2018.01120 30337875 PMC6178849

[B20] Li F. Zhang Y. Wang Z.-H. Gao S. Fan Y.-C. Wang K. (2023). SOCS1 methylation level is associated with prognosis in patients with acute-on-chronic hepatitis B liver failure. Clin. Epigenetics 15 (1), 79. 10.1186/s13148-023-01495-9 37149648 PMC10163770

[B21] Li J. Liang X. Jiang J. Yang L. Xin J. Shi D. (2022). PBMC transcriptomics identifies immune-metabolism disorder during the development of HBV-ACLF. Gut 71 (1), 163–175. 10.1136/gutjnl-2020-323395 33431576 PMC8666828

[B22] Li L.-C. Dahiya R. (2002). MethPrimer: designing primers for methylation PCRs. Bioinformatics 18 (11), 1427–1431. 10.1093/bioinformatics/18.11.1427 12424112

[B23] Liu F. Zou Z. Shen L. Wu W. Luo J. Lankford S. (2020). A prediction model for outcome in patients with HBV-ACLF based on predisposition, injury, response and organ failure. Sci. Rep. 10 (1), 20176. 10.1038/s41598-020-77235-3 33214662 PMC7677318

[B24] Liu G. Z. Xu X. W. Tao S. H. Gao M. J. Hou Z. H. (2021). HBx facilitates ferroptosis in acute liver failure via EZH2 mediated SLC7A11 suppression. J. Biomed. Sci. 28 (1), 67. 10.1186/s12929-021-00762-2 34615538 PMC8495979

[B25] Liu K. Liu J. Zou B. Li C. Zeh H. J. Kang R. (2022). Trypsin-mediated sensitization to ferroptosis increases the severity of pancreatitis in mice. Cell Mol. Gastroenterol. Hepatol. 13 (2), 483–500. 10.1016/j.jcmgh.2021.09.008 34562639 PMC8688567

[B26] Malinchoc M. Kamath P. S. Gordon F. D. Peine C. J. Rank J. ter Borg P. C. (2000). A model to predict poor survival in patients undergoing transjugular intrahepatic portosystemic shunts. Hepatology 31 (4), 864–871. 10.1053/he.2000.5852 10733541

[B27] Mayr L. Grabherr F. Schwärzler J. Reitmeier I. Sommer F. Gehmacher T. (2020). Dietary lipids fuel GPX4-restricted enteritis resembling Crohn’s disease. Nat. Commun. 11 (1), 1775. 10.1038/s41467-020-15646-6 32286299 PMC7156516

[B28] Nie Y. Zhang Y. Liu L. X. Zhu X. (2020). Serum lactate level predicts short-term and long-term mortality of HBV-ACLF patients: a prospective study. Ther. Clin. Risk Manag. 16, 849–860. 10.2147/tcrm.S272463 32982257 PMC7490053

[B29] Piano S. Mahmud N. Caraceni P. Tonon M. Mookerjee R. P. (2023). Mechanisms and treatment approaches for ACLF. Liver Int. 10.1111/liv.15733 PMC12036731 37715608

[B30] Sarin S. K. Choudhury A. (2016). Acute-on-chronic liver failure: terminology, mechanisms and management. Nat. Rev. Gastroenterol. Hepatol. 13 (3), 131–149. 10.1038/nrgastro.2015.219 26837712

[B31] Sarin S. K. Choudhury A. Sharma M. K. Maiwall R. Al Mahtab M. Rahman S. (2019a). Acute-on-chronic liver failure: consensus recommendations of the Asian Pacific association for the study of the liver (APASL): an update. Hepatol. Int. 13 (4), 353–390. 10.1007/s12072-019-09946-3 31172417 PMC6728300

[B32] Sengupta A. Lichti U. F. Carlson B. A. Cataisson C. Ryscavage A. O. Mikulec C. (2013). Targeted disruption of glutathione peroxidase 4 in mouse skin epithelial cells impairs postnatal hair follicle morphogenesis that is partially rescued through inhibition of COX-2. J. Invest Dermatol 133 (7), 1731–1741. 10.1038/jid.2013.52 23364477 PMC3652900

[B33] Sun L. Li K. Liu G. Xu Y. Zhang A. Lin D. (2018). Distinctive pattern of AHNAK methylation level in peripheral blood mononuclear cells and the association with HBV-related liver diseases. Cancer Med. 7 (10), 5178–5186. 10.1002/cam4.1778 30259695 PMC6198198

[B34] Sun L. Lu J. Li K. Zhang H. Zhao X. Li G. (2022). Diagnostic and prognostic value of STAP1 and AHNAK methylation in peripheral blood immune cells for HBV-related hepatopathy. Front. Immunol. 13, 1091103. 10.3389/fimmu.2022.1091103 36713363 PMC9880311

[B35] Terrault N. A. Lok A. S. F. McMahon B. J. Chang K. M. Hwang J. P. Jonas M. M. (2018). Update on prevention, diagnosis, and treatment of chronic hepatitis B: AASLD 2018 hepatitis B guidance. Hepatology 67 (4), 1560–1599. 10.1002/hep.29800 29405329 PMC5975958

[B36] Tong J. Li D. Meng H. Sun D. Lan X. Ni M. (2022). Targeting a novel inducible GPX4 alternative isoform to alleviate ferroptosis and treat metabolic-associated fatty liver disease. Acta Pharm. Sin. B 12 (9), 3650–3666. 10.1016/j.apsb.2022.02.003 36176906 PMC9513461

[B37] Ursini F. Maiorino M. Forman H. J. (2016). Redox homeostasis: the Golden Mean of healthy living. Redox Biol. 8, 205–215. 10.1016/j.redox.2016.01.010 26820564 PMC4732014

[B38] Wang F. Sun W. Xiao Q. Liang C. Jiang S. Lian Y. (2021). Peripheral T lymphocytes predict the severity and prognosis in patients with HBV-related acute-on-chronic liver failure. Med. Baltim. 100 (5), e24075. 10.1097/md.0000000000024075 PMC7870253 33592861

[B39] Wang Q. Bin C. Xue Q. Gao Q. Huang A. Wang K. (2021). GSTZ1 sensitizes hepatocellular carcinoma cells to sorafenib-induced ferroptosis via inhibition of NRF2/GPX4 axis. Cell Death Dis. 12 (5), 426. 10.1038/s41419-021-03718-4 33931597 PMC8087704

[B40] Weaver K. Skouta R. (2022). The selenoprotein glutathione peroxidase 4: from molecular mechanisms to novel therapeutic opportunities. Biomedicines 10 (4), 891. 10.3390/biomedicines10040891 35453641 PMC9027222

[B41] Wei X.-F. Zhu J.-Y. Liu H.-H. Su X. Li J.-H. Fan Y.-C. (2023). Hypomethylation of Tumor necrosis factor-like cytokine 1A(TL1A) and its decoy receptor 3 expressive level increase has diagnostic value in HBV-associated cirrhosis. Virology 585, 91–99. 10.1016/j.virol.2023.04.009 37321146

[B42] Xie Y. Kang R. Klionsky D. J. Tang D. (2023). GPX4 in cell death, autophagy, and disease. Autophagy 19 (10), 2621–2638. 10.1080/15548627.2023.2218764 37272058 PMC10472888

[B43] Xu S. Li X. Zhang S. Qi C. Zhang Z. Ma R. (2023). Oxidative stress gene expression, DNA methylation, and gut microbiota interaction trigger Crohn’s disease: a multi-omics Mendelian randomization study. BMC Med. 21 (1), 179. 10.1186/s12916-023-02878-8 37170220 PMC10173549

[B44] Xue R. Yang J. Wu J. Wang Z. Meng Q. (2021). Novel prognostic models for predicting the 180-day outcome for patients with hepatitis-B virus-related acute-on-chronic liver failure. J. Clin. Transl. Hepatol. 9 (4), 514–520. 10.14218/jcth.2021.00028 34447680 PMC8369019

[B45] Yang L. Zou T. Chen Y. Zhao Y. Wu X. Li M. (2022). Hepatitis B virus X protein mediated epigenetic alterations in the pathogenesis of hepatocellular carcinoma. Hepatol. Int. 16 (4), 741–754. 10.1007/s12072-022-10351-6 35648301

[B46] Yang W. Wang Y. Zhang C. Huang Y. Yu J. Shi L. (2022). Maresin1 protect against ferroptosis-induced liver injury through ROS inhibition and Nrf2/HO-1/GPX4 activation. Front. Pharmacol. 13, 865689. 10.3389/fphar.2022.865689 35444546 PMC9013935

[B47] Yang W. S. SriRamaratnam R. Welsch M. E. Shimada K. Skouta R. Viswanathan V. S. (2014). Regulation of ferroptotic cancer cell death by GPX4. Cell 156 (1-2), 317–331. 10.1016/j.cell.2013.12.010 24439385 PMC4076414

[B48] Yuan L. Li S. Chen Q. Xia T. Luo D. Li L. (2022). EBV infection-induced GPX4 promotes chemoresistance and tumor progression in nasopharyngeal carcinoma. Cell Death Differ. 29 (8), 1513–1527. 10.1038/s41418-022-00939-8 35105963 PMC9346003

[B49] Zaccherini G. Weiss E. Moreau R. (2021). Acute-on-chronic liver failure: definitions, pathophysiology and principles of treatment. JHEP Rep. 3 (1), 100176. 10.1016/j.jhepr.2020.100176 33205036 PMC7652714

