# Supplementary figures and images for: Hypermethylation of the glutathione peroxidase 4 promoter predicts poor prognosis in patients with hepatitis B virus-associated acute-on-chronic liver failure
Source: Front Mol Biosci. 2024 Jul 25;11:1421597. doi: 10.3389/fmolb.2024.1421597 (PMC11306020; doi:10.3389/fmolb.2024.1421597)

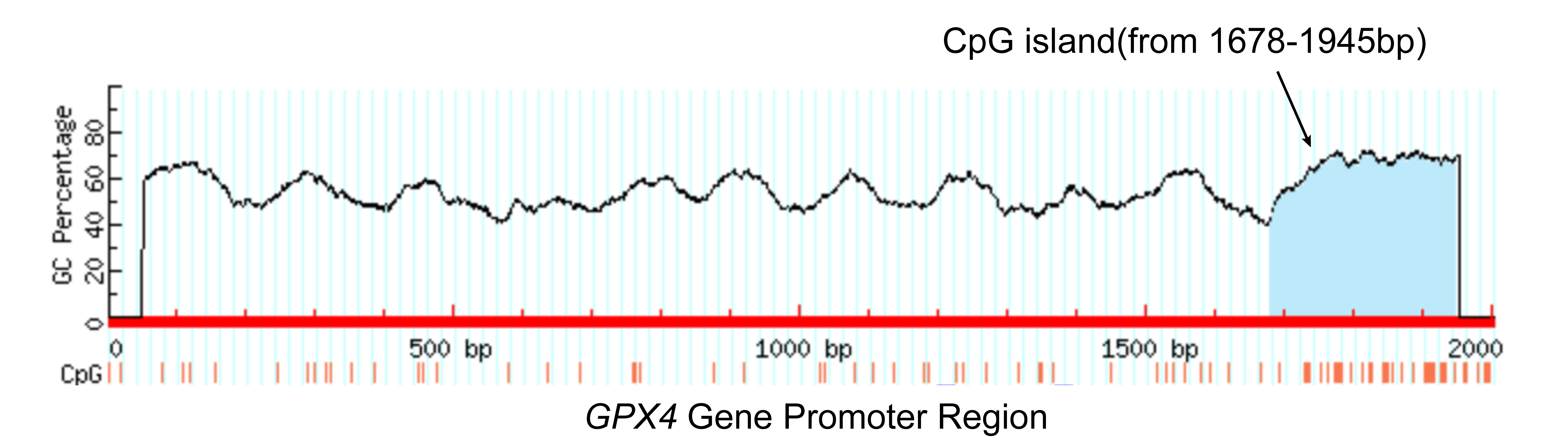

Supplement: Supplementary file 1 [file Image1.TIF]
